# An Ultra High-Throughput, Whole-Animal Screen for Small Molecule Modulators of a Specific Genetic Pathway in *Caenorhabditis elegans*

**DOI:** 10.1371/journal.pone.0062166

**Published:** 2013-04-29

**Authors:** Chi K. Leung, Ying Wang, Siobhan Malany, Andrew Deonarine, Kevin Nguyen, Stefan Vasile, Keith P. Choe

**Affiliations:** 1 Department of Biology and Genetics Institute, University of Florida, Gainesville, Florida, United States of America; 2 Sanford-Burnham Medical Research Institute at Lake Nona, Orlando, Florida, United States of America; University of Pennsylvania, United States of America

## Abstract

High-throughput screening (HTS) is a powerful approach to drug discovery, but many lead compounds are found to be unsuitable for use *in vivo* after initial screening. Screening in small animals like *C. elegans* can help avoid these problems, but this system has been limited to screens with low-throughput or no specific molecular target. We report the first *in vivo* 1536-well plate assay for a specific genetic pathway in *C. elegans*. Our assay measures induction of a gene regulated by SKN-1, a master regulator of detoxification genes. SKN-1 inhibitors will be used to study and potentially reverse multidrug resistance in parasitic nematodes. Screens of two small commercial libraries and the full Molecular Libraries Small Molecule Repository (MLSMR) of ∼364,000 compounds validate our platform for ultra HTS. Our platform overcomes current limitations of many whole-animal screens and can be widely adopted for other inducible genetic pathways in nematodes and humans.

## Introduction

Advances in chemistry and robotics have resulted in enormous small molecule libraries (>1 million compounds) available for high-throughput screening [1]. *In vitro* and cell-based assays are specific approaches for identifying preliminary hits [2], [3], [4]. However, after expensive and laborious follow-up studies in whole-animals, hit compounds are often found to be inappropriate for *in vivo* use due to unfavorable drug absorption, distribution, metabolism, excretion, and toxicity (pharmacokinetics), known as ADMET [5], [6], [7], [8]. Compounds may also lack specificity or have synergistic effects compromising safety. Screening in whole-animals can help overcome some of these pitfalls in drug discovery from the onset of screening [9]. The model nematode *C. elegans* is well-suited to high-throughput, whole-animal screening [10], [11], [12], [13]. *C. elegans* is free-living, small (≤1 mm), inexpensive to culture, has a short life-cycle and high fecundity, and is transparent at all developmental stages facilitating the use of fluorescent probes to monitor cellular processes *in vivo*. *C. elegans* is also one of the most experimentally tractable model animals and there is a tremendous wealth of knowledge on biology and genetics of this organism [14], [15], [16], [17]. The genetic tractability of *C. elegans* can be exploited to rapidly define mode of action for pharmacological compounds [18], [19], [20], [21], [22]. Furthermore, *C. elegans* has been, and will continue to be, an important model for basic nematode biology and anthelmintic discovery [9], [23], [24], [25].

Phenotype-based whole-animal assays have been developed to facilitate drug discovery and drug target identification by monitoring locomotion, behavior, morphology, feeding, brood size, longevity, and development [12], [26], [27], [28], [29], [30]. However, scoring of these assays is either labor intensive or technically demanding limiting them to low- to medium-throughput. In addition, multiple signaling pathways contributing to a complex phenotype may be targeted making it difficult to define mode-of-action and single pathways of interest [31], [32]. *In vivo* fluorescent reporters for specific genetic pathways, which are routine in *C. elegans* [33], [34], have the potential to overcome many of these problems and fit well into the current pipelines of modern HTS facilities.

Nematode parasites cause an estimated $80 billion loss of food production each year [35], are a major burden to animal husbandry, and infect as many as 1/3 of humans world-wide [36], [37]. Multidrug resistance is a growing problem in parasitic nematodes, and mechanisms of resistance are poorly understood in this group of animals in part because genetic tractability is limited [38]. In systems ranging from microbes to cancer cells, multidrug resistance is mediated by increased expression and activity of enzymes that detoxify xenobiotics [39], [40], [41], [42], [43], [44].

Inducible cap’n’collar transcription factors (CNCs) are master regulators of detoxification, antioxidant, and cellular repair genes in animals and function to promote drug and xenobiotic detoxification, stress resistance, and extend lifespan [45]. Increased activity of Nrf2, the mammalian CNC, has been shown to mediate multidrug resistance in human tumors [46], [47], [48], [49]. SKN-1, the *C. elegans* CNC homolog, orchestrates the transcriptional response to oxidants and electrophilic xenobiotics [50], [51], [52]. SKN-1 is also required for specification of pharyngeal and intestinal tissues during embryogenesis, and loss of *skn-1* results in embryonic lethality [53]. Genes families regulated by SKN-1 have also been implicated in drug resistance in other nematode species [38]. We previously defined a principal repressor of SKN-1 that is molecularly distinct from the pathway that regulates Nrf2 in mammals [54]. SKN-1 also binds to DNA by a unique monomeric mechanism relative to other basic leucine zipper factors [55]. These unique features of SKN-1 structure and regulation could serve as targets for small molecule inhibitors. Pharmacological compounds that target SKN-1 would provide new tools to study the function of the inducible antioxidant and detoxification response in medically and agriculturally important non-model nematodes and have the potential to inhibit embryonic development, reverse drug resistance, and increase the useful life of current and future anthelmintics [38].

We recently developed a genetically encoded, dual fluorescence-based assay for a core SKN-1 regulated gene, *gst-4* [56]. Here, we optimize this assay for 1536-well microplate format, perform pilot screens of two commercially available libraries, develop a counter-screen and manual secondary assays to identify off-target mechanisms, and perform a primary uHTS screen of the entire NIH Molecular Libraries Small Molecule Repository (MLSMR) of ∼364,000 compounds. Throughput and performance of our *in vivo* assay was comparable to cell-based assays and many lead compounds were identified providing proof-of-principle that our approach can become a robust addition to the ultra HTS drug discovery toolkit.

## Materials and Methods

### *C. elegans* Strains and Transgenes

The following strains were used: wild-type N2 Bristol, CL2166 dvIs19[pAF15(Pgst-4::GFP::NLS)], VP596 dvIs19[pAF15(Pgst-4::GFP::NLS)];vsIs33[Pdop-3::RFP], TJ375 gpIs[Phsp-16.2::GFP], QV65 gpIs[Phsp-16.2::GFP];vsIs33[Pdop-3::RFP], and QV63 zjEx38[Pvha-6::GFP];unc-119(ed3). Unless noted otherwise, worms were cultured at 20°C using standard methods [57].

### Preparation of Bacteria, Worms, and Reagents

OP50 bacteria culture, large-scale liquid worm culture, and dispensing of worms for HTS were performed as described recently [56] and briefly summarized as follows. An overnight culture of OP50 bacteria grown in terrific broth was washed and resuspended in an equal volume of nematode growth medium (NGM) buffer to prepare 50% concentrated OP50 stocks, which were stored at −20°C. Worms were synchronized using the standard hypochlorite procedure and approximately two million eggs were shaken in a flask at 100 rpm at 20°C in NGM buffer. The next day, a frozen stock of 50% OP50 bacteria culture was thawed and added to the synchronized L1 larvae. During worm growth, bacteria were added to keep OD600 above 0.9. Worms were grown for approximately 51 hours, or until they developed to the L4 larval and young adult stages. To prepare for dispensing into microplates, worms were washed thoroughly with NGM buffer containing 1% Luria broth (three to four times) to remove bacteria.

Juglone (5-Hydroxy-p-naphthoquinone, CAS 481390, Acros Organics, Belgium, New Jersey) was first dissolved in DMSO at 100 mM and stored at −20°C; final juglone solutions were prepared fresh by diluting the stock in NGM buffer [56]. Acrylamide (Sigma, A8887, St. Louis, MO) was dissolved in NGM buffer and filtered-sterilized.

### SPECTRUM, LOPAC, and MLSMR Assay Plates

The SPECTRUM Collection (Microsource Discovery Systems, Gaylordsville, CT) of diverse compounds was obtained from the Vanderbilt University Medical Center screening facility and screened in 384 black well plates (Greiner Bio-One, Monroe, NC) at final concentrations of 10, 20, or 40 µM. The first and last two columns of each plate were used for un-induced and induced controls. Twenty-five µl of 60–75 L4 larval to young adult stage worms were dispensed into all wells using a BioTek Microflo Select with a 10 µl dispensing cassette as described previously [56]. Worms were incubated with the SPECTRUM compounds for 1.5 h. Twenty-five µl of NGM buffer with juglone (38 µM final concentration) were then dispensed using a 1 µl Microflo dispensing cassette.

The Library of Pharmacologically Active Compounds (LOPAC) was purchased from Sigma-Aldrich (St. Louis, MO). The test compounds (in DMSO) or DMSO were transferred to columns 5–48 and 1–4, respectively of black HiBAse 1536-well plates (Aurora, Carlsbad, CA), using a Labcyte ECHO 555 (Labcyte Inc., Sunnyvale, CA). Five µl of 30–35 L4 larval to young adult stage worms were dispensed into all wells using a 5 µl Microflo dispensing cassette. Worms were incubated with the LOPAC compounds for 1.5 h. Three µl of NGM buffer and acrylamide (final concentration 7 mM) were dispensed into columns 1–4 and 5–48, respectively using a 1 µl Microflo dispensing cassette. A primary screen of the MLSMR library of ∼364,000 compounds was performed with the same parameters used for the LOPAC screen.

### Microscopy

Fluorescent and differential interference contrast images were captured with an Olympus BX60 microscope (Olympus, Melville, NY) with UPlanFL 4X/0.13, 10X/0.30, 20X/0.50, 40X/0.75 or 60X/1.25 oil lenses and a Zeiss Axiocam MRm camera (Carl Zeiss, Thornwood, NY).

### Quantitative PCR

Quantitative real-time RT-PCR was used to measure mRNA levels in L4 larval to young adult stage worms as described previously [54], [58], [59]. Total RNA was isolated from drug-treated worms with an RNAqueous-Micro kit (Ambion, Austin, TX), and cDNA was synthesized using 1 µg of RNA with Superscript III following the manufacturer’s protocol (Invitrogen, Carlsbad, CA). Quantitative real-time PCR was conducted in a Realplex ep gradient S Mastercycler (Eppendorf AG, Hamburg, Germany). Four µl of each cDNA were amplified in a total volume of 10 µl with GoTaq Green Master Mix (Promega, Madison, WI) according to the manufacturer’s protocol. The housekeeping ribosomal protein gene *rpl-2* was used as an internal control; we have observed stable expression of this mRNA in worms exposed to a variety of stressors [54], [58], [60]. Melting curves for each PCR were carefully monitored to avoid nonspecific amplifications. Primer sequences are available upon request.

### HTS Data Acquisition

All the fluorescence analyses in 384 well plates, including the SPECTRUM Library screen, were done as previously described [56]. Fluorescence in the LOPAC and NIH screen using 1536 well plates was measured with an EnVision MultiLabel® Plate reader (PerkinElmer Life Sciences, Boston, MA) with the following settings: GFP - FITC485/14_ex_ FITC535/25_em_; RFP - BODIPY TMR FP 531/25_ex_ Cy3 595/60_em_. The fluorescence signals were collected between 20 and 24 hours after addition of juglone or acrylamide. The GFP/RFP ratio was calculated and normalized to the fluorescence reading of the controls wells (without SKN-1 inducers) to determine the fold change of fluorescence intensity derived from individual treatments. To eliminate toxic or autofluorescent compounds, we limited hits to RFP values within three standard deviations or within 50% of the mean RFP fluorescence of control wells on the same plates in the SPECTRUM and LOPAC (and NIH) screens, respectively. Z-factors for individual assays were calculated as 1–3(σ_p_+σ_n_)/(µ_p_-µ_n_), where σ is the standard deviation, µ is the mean, *p* is positive control (with SKN-1 inducers), and *n* is negative control (without SKN-1 inducers).

### Stress Resistance Assays

Fifty µl of 15–20 synchronized L4 larval to young adult stage N2 worms were dispensed into clear 96 well microtiter plates. Worms were then exposed to compounds for 1.5 h followed by juglone (225 µM) or acrylamide (2.8 mM) at 20°C. The number of dead worms in each well were counted at 0, 2, 4, and 6 h for juglone or 20 h for acrylamide. Worms were considered alive if they displayed any movement in response to repeated prodding with a thin wire or tapping of plates.

### Heat-shock Counter Screen

In heat-shock assays, synchronized populations of L4 larval to young adult stage QV65 worms were incubated with compounds at 20°C for 1.5 h in 384 well plates. The plates were then shifted to 35°C for 1 h followed by 5 h of recovery at 20°C before the fluorescence was measured as described previously [56].

### Statistical Analysis

Statistical significance was determined using one-way analysis of variance with a Dunnet or Tukey’s post-hoc test when three or more means were compared and two-way analysis of variance with a Bonferonni post-hoc test when means were compared across two factors. Log-rank tests with Bonferonni correction were used to compare survival curves. *P* values of <0.05 were taken to indicate statistical significance. IC_50_ values were calculated from eight-point dose-response curves drawn from ten replicates, using nonlinear regression curves (PRISM 5.0, GraphPad Software, San Diego, CA).

## Results

### SPECTRUM Library Screen

We previously demonstrated that the napthoquinone juglone can strongly activate detoxification genes via *skn-1* [54]. We also developed protocols for large scale liquid-based worm culture and dispensing into 384-well plates to monitor SKN-1 activity *in vivo* [56]. The assay measures the activity of SKN-1 by monitoring the fluorescence of a GFP reporter driven by the promoter of a well-established glutathione S-transferase gene target regulated by SKN-1, *gst-4* (*Pgst-4::GFP*) [51], [52], [54] relative to fluorescence of a constitutively expressed RFP reporter (*Pdop-3::RFP*) used to normalize for variation of worm number. Glutathione S-transferases are a large class of detoxification enzymes that catalyze the conjugation of glutathione to electrophiles. In *C. elegans*, expression of *gst-4* is activated by redox cycling compounds, electrophiles, and heavy metals [56], [61], [62] and *gst-4* is required for resistance to juglone [63]. We recently demonstrated that *Pgst-4::GFP* induction by juglone is dose-dependent, tolerant of DMSO, stable over several hours, and robust in 384-well plates [56].

Here, we used this assay to run a pilot screen of the SPECTRUM collection, which contains 2320 biologically active and structurally diverse compounds. We tested the inhibitory effects of these small molecules on *Pgst-4::GFP* induction at 10, 20, and 40 µM using 38 µM juglone as a SKN-1 inducer. The first and last two columns of each plate served as un-induced (0.038% DMSO) and induced (38 µM juglone) controls. VP596 worms were dispensed into individual 384-well plates (7 plates per concentration) and then 38 µM juglone was added after 1.5 h at 20°C. GFP and RFP fluorescence were then measured after 21 h. The normalized fold induction of GFP/RFP ratios, Z-factors, and number of hits according to their % inhibition in each concentration are summarized in Table 1 and all hit compounds are listed in Table S1 in File S1. We limited hits to compounds that had an RFP value within three standard deviations of the mean RFP fluorescence of un-induced wells on the same plate to eliminate false-positive compounds that were toxic or that were fluorescent in the red channel.

**Table 1:** SPECTRUM screening summary in 384-well plates.

			Number of Hits		
	S/B	Z-factor	>60%	>50%	>40%
10 µM	11.47±0.56	0.58±0.06	1	1	6
20 µM	23.04±1.38	0.71±0.03	1	3	28
40 µM	9.22±0.97	0.54±0.06	0	4	26

S/B = signal to background. Hits are expressed as % inhibition. S/B and Z-factors were calculated by averaging the S/B and Z-factors in seven plates per concentration. *n = *32 wells per treatment.

Phenylmercuric acetate, fluorescein, phloretin, and phloroacetophenone were the only compounds that inhibited *Pgst-4::GFP* fluorescence over 40% at all concentrations. Phenylmercuric acetate is known to be highly toxic and fluorescein is a fluorophore with excitation and emission spectra that overlap with RFP. Therefore, these compounds were considered artifacts. Phloroacetophenone and phloretin were characterized further (see below).

### Acrylamide is a more Stable and Stronger gst-4 Inducer than Iuglone

We often observed inconsistent levels of *Pgst-4::GFP* activation and Z-factors between different plates and days in the assay using juglone as a SKN-1 inducer (Table 1 and unpublished data). This variability may be caused by rapid degradation of juglone, which is unstable in near neutral or alkaline solutions at room temperature [64], [65]. Early during assay optimization, we also had problems with juglone binding or reacting with tubing in dispensing cassettes resulting in a gradient of reporter induction within plates (unpublished data). Therefore, we searched for a more stable and consistent SKN-1 inducer.

**Figure 1:**
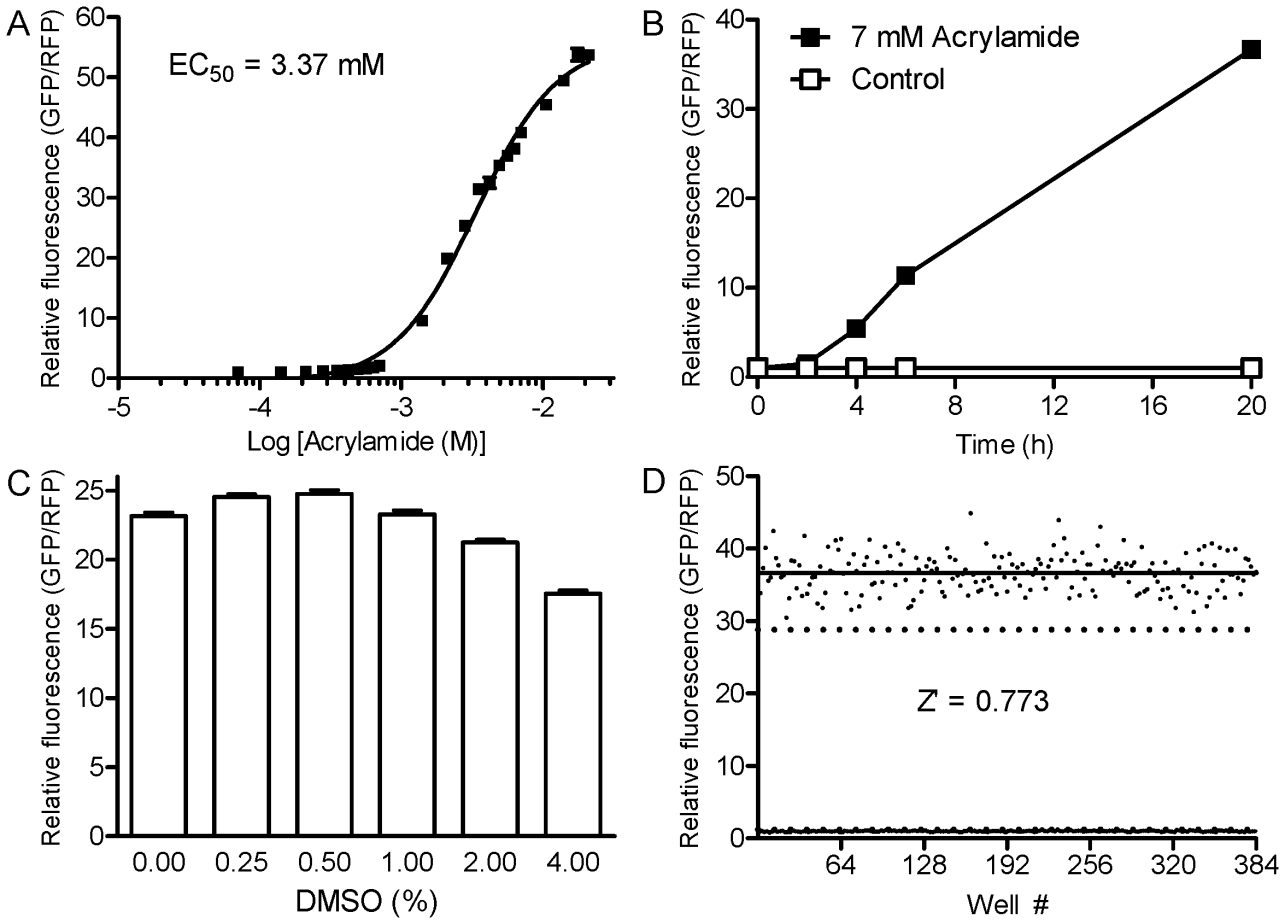
Activation of *Pgst-4::GFP* by acrylamide is robust and consistent. L4 larval to young adult stage worms were diluted to approximately 2–3/µl and 30 µl was dispensed into every well of a 384 well plate. GFP and RFP fluorescence were measured after 21 h. (A) Fluorescence was measured with different concentrations of acrylamide (*n = *16 wells). (B) Seven mM acrylamide was added in every other column to measure fluorescence at 0, 2, 4, 6 and 20 h (*n = *192 wells). (C) Fluorescence was measured with 7 mM acrylamide and different concentrations of DMSO (*n = *64 wells). 2 and 4% DMSO decreased induction relative to control, *P*<0.001. (D) Assay consistency is shown using a scatter plot to compare the fluorescence of acrylamide-treated (7 mM) worms to that of control worms (*n = *192 wells per treatments). The mean relative fluorescence ratios of all control (1.0) and acrylamide wells (36.7) are marked with solid lines. Three standard deviations above the control mean and below the acrylamide mean are marked with broken lines. Values are means ± SEM (A–C) or individual data points (D).

Acrylamide was recently shown to strongly induce *Pgst-4::GFP via* SKN-1 with low toxicity in *C. elegans* [62], [66], [67]. Figure 1A shows that *Pgst-4::GFP* induction is dose-dependent with acrylamide in 384-well plates with an EC_50_ of 3.37 mM. Figure 1B shows that *Pgst-4::GFP* was induced by 10-fold or more between 8 and 20 h; induction remained greater than 10-fold for at least 36 h (data not shown). Acrylamide-induced *Pgst-4::GFP* expression did not decrease in the presence of DMSO up to 1%; it did decrease by 8.3 and 24.2% in 2 and 4% DMSO, respectively (Figure 1C, *P*<0.001). The assay was robust with a Z-factor of 0.773 (Figure 1D). We also observed no left to right gradient of *Pgst-4::GFP* induction within 384-well plates. In five full plates with alternating columns of vehicle (NGM buffer) and acrylamide (7 mM) run on different days, the Z-factors ranged from 0.708 to 0.774 (mean of 0.737±0.012) and the fold-induction of GFP/RFP ratio ranged from 33.4 to 34.8. Therefore, acrylamide is a more stable and consistent SKN-1 activator than juglone. The concentration of acrylamide needed to strongly activate *Pgst-4::GFP* is high, but acrylamide is inexpensive and highly soluble in NGM buffer. We also expect that using a high concentration of SKN-1 inducer will reduce the number of false-positive hits that simply react with the inducer outside of worms, because acrylamide will be present at over 300-fold molar excess relative to library compounds tested at 20 µM.

### Optimization of the SKN-1 Assay in 1536-well Plates

To increase throughput, we tested our assay in 1536-well plates using acrylamide. We optimized the number of worms dispensed in each well based on the fold induction of GFP/RFP ratios and Z-factors as summarized in Table S2 in File S1. VP596 worms were diluted at different densities and dispensed into all wells of 1536-well plates. Seven mM acrylamide and NGM buffer (vehicle) were added in alternating columns. GFP and RFP fluorescence were measured after 20 h at 20°C. Visual inspection indicated that worms were viable in 1536-well plates for at least 24 h. A density of 30–35 worms per well resulted in the highest Z-factor of 0.72 with a signal to background ratio of 18.92±1.47 (Table S2 in File S1). It is worth noting that the signal to background ratio obtained in 1536-well plates was almost half that of 384-well plates regardless of the number of worms in each well, suggesting that *Pgst-4::GFP* induction is somewhat attenuated in the small dimensions of a 1536-well plate. Nonetheless, acrylamide-induced *Pgst-4::GFP* expression was robust and reproducible in a 1536-well plates.

### LOPAC Screen

**Figure 2:**
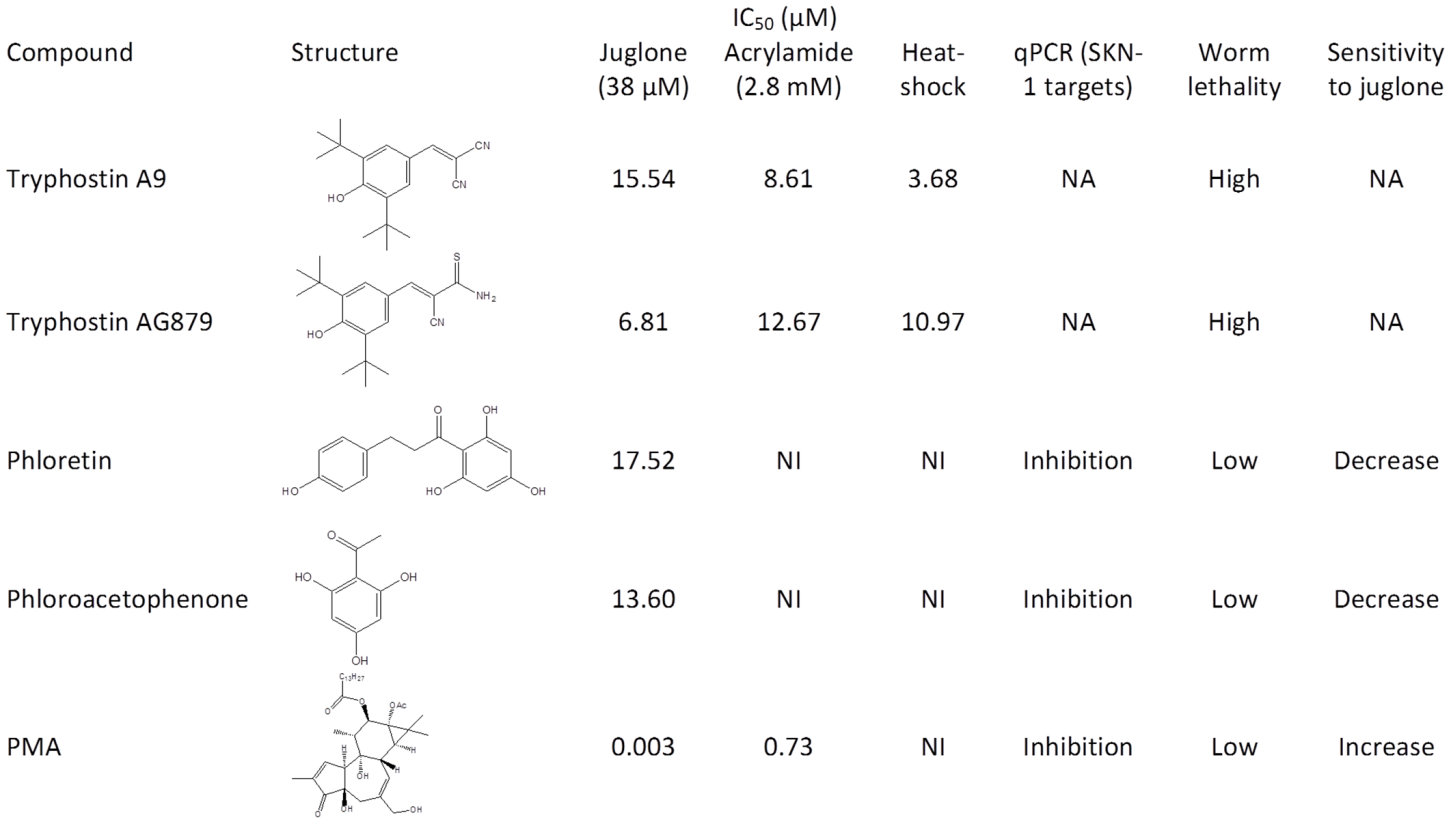
Summary of hit compounds that were characterized in this study. Structures were generated by CambridgeSoft ChemDraw Ultra 7.0 (Cambridge, MA). NI = No Inhibition. NA = Not Applicable.

Given that acrylamide is more convenient to use and provides more robust and consistent *Pgst-4::GFP* reporter activation than juglone, we used it to perform a screen of the LOPAC compound library in 1536-well plates. The LOPAC library contains 1280 pharmacologically active compounds targeting many cellular processes and covering all major drug target classes. We ran the LOPAC screen at 5, 10, and 20 µM in triplicate for each concentration. The number of hits, the signal to background ratios of GFP/RFP, and Z-factors for individual plates are summarized in Table 2, and all hit compounds are listed in Table S3 in File S1. Tyrphostin A9, tyrphostin AG879, and phorbol 12-myristate 13-acetate (PMA) consistently inhibited >40% of GFP/RFP induction at all three concentrations and were characterized further. The results for all five compounds characterized from the SPECTRUM and LOPAC screens are summarized in Figure 2.

**Table 2:** LOPAC screening summary in 1536-well plates.

			Number of Hits		
	S/B	Z-factor	>60%	>50%	>40%
5 µM (0.25% DMSO)					
Replicate 1	18.7	0.70	5	8	9
Replicate 2	21.1	0.72	3	6	10
Replicate 3	20.3	0.71	6	6	7
10 µM (0.25% DMSO)					
Replicate 1	19.0	0.68	4	6	9
Replicate 2	21.0	0.71	4	7	11
Replicate 3	19.0	0.71	6	9	9
20 µM (0.25% DMSO)					
Replicate 1	20.0	0.69	4	9	13
Replicate 2	20.3	0.69	6	9	14
Replicate 3	19.5	0.72	7	10	16

S/B = signal to background. Hits are expressed as % inhibition. *n* = 32 wells per treatment.

### Dose-dependent Inhibition of Pgst-4::GFP Induction

**Figure 3:**
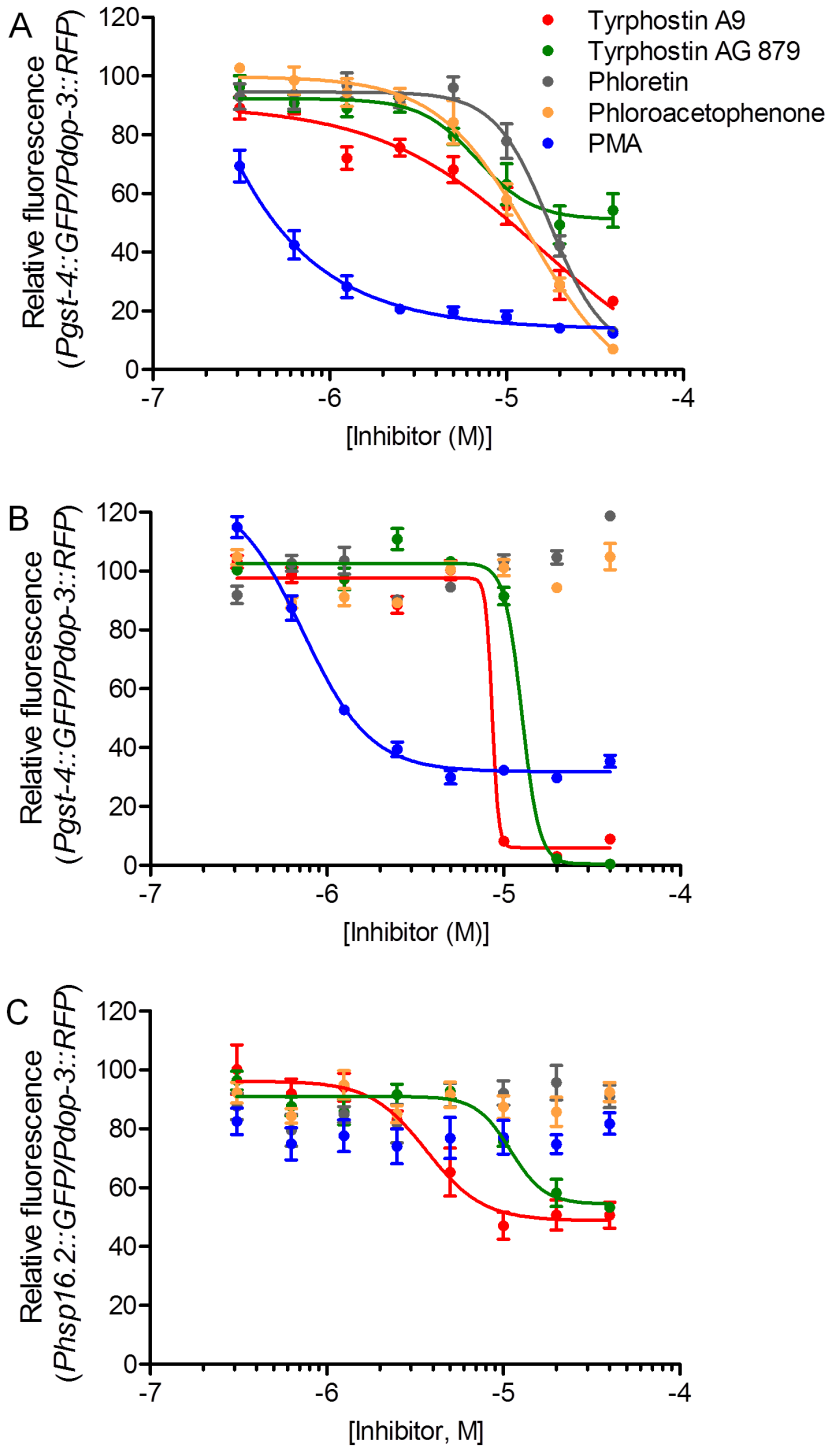
Dose-dependent inhibition of fluorescent reporters. *Pgst-4::GFP* and *Pdop-3::RFP* fluorescence was measured after 21 h exposure to 38 µM juglone (A) or 2.8 mM acrylamide (B) in 384 well plates (*n = *32 wells). (C) *Phsp-16.2::GFP* and *Pdop-3::RFP-*expressing worms were heat-shocked at 35°C for 1 h followed by 5 h recovery at 20°C (*n = *32 wells). Values are means ± SEM.

Tyrphostin A9, tyrphostin AG879, and PMA from the LOPAC screen, and phloroacetophenone and phloretin from the SPECTRUM screen, were further analyzed at multiple doses. The three compounds from the LOPAC library screen all caused a dose-dependent inhibition of GFP/RFP induction by juglone and acrylamide (Figures 3A and B). Alternatively, the two compounds from the SPECTRUM library only inhibited GFP/RFP induction by juglone, indicating that the mechanism of SKN-1 inhibition for these two related compounds, phloroacetophenone and phloretin, is specific to juglone. IC_50_ values are listed in Figure 2.

### Heat-shock Response Counter Screen

We expect that many compounds could non-specifically inhibit *Pgst-4::GFP* induction due to general effects on transcription, translation, or cytotoxicity. To eliminate these off-target hits, we developed a new fluorescent reporter strain based on a separate inducible pathway. The heat-shock factor HSF-1 is activated independently from SKN-1 during thermal stress and activates the expression of a suite of protein folding chaperones including *hsp-16.2* [68]. Previous studies have demonstrated that the *Phsp-16.2::GFP* reporter is activated robustly following heat-shock at 35°C for 1 h and that the signal is stable between 4 and 24 h [69].

We first crossed the *Pdop-3::RFP* normalization reporter with the *Phsp-16.2::GFP* reporter to create the new strain, QV65. We tested the induction of *Phsp-16.2::GFP* by dispensing 50–70 QV65 worms into each well of a 384-well plate. The plate was shifted to 35°C for 1 or 2 h followed by recovery at 20°C and GFP and RFP fluorescence was measured at multiple time points. Maximal induction of *Phsp-16.2::GFP* was achieved with a 1 h heat-shock at 35°C followed by 5 h recovery at 20°C (Figure S1A). The fold induction was ∼5.6 fold with a Z–factor of ∼0.70. *Phsp-16.2::GFP* induction was also DMSO-tolerant (Figure S1B); reporter activation decreased by only 4% from 0 to 1% DMSO and decreased by 16.0% and 28.1% in 2 and 4% DMSO, respectively (*P*<0.001).

We tested the five hit compounds listed in Figure 2 for dose-dependent inhibition of the heat-shock response reporter. As shown in Figure 3C, the two related compounds from the LOPAC library, tyrphostin A9 and tyrphostin AG879, inhibited *Phsp-16.2::GFP* induction indicating that they are not specific to SKN-1. None of the other compounds inhibited the heat-shock response suggesting that their mechanisms of inhibition are more specific.

### Suppression of SKN-1-dependent Detoxification Genes

Given that tyrphostin A9 and tyrphostin AG879 were non-specific, we focused further characterization on the other three compounds. To determine if the other compounds suppress activation of endogenous detoxification genes regulated by SKN-1, we performed quantitative real-time RT-PCR for multiple genes regulated by *skn-1* [51], [52]. Wildtype N2 worms were treated with 20 µM of each compound for 1.5 h at 20°C followed by 4 h with 2.8 mM acrylamide for PMA or 38 µM juglone for phloroacetophenone and phloretin. PMA reduced induction of five out of seven genes tested (Figure S2A). Phloroacetophenone and phloretin reduced induction of all seven SKN-1 target genes tested. Taken together, the real-time RT-PCR results demonstrate that our fluorescence reporter-based *in vivo* assay is able to identify compounds that inhibit endogenous SKN-1 dependent genes.

### Toxicity and Oxidative Stress Resistance

**Figure 4:**
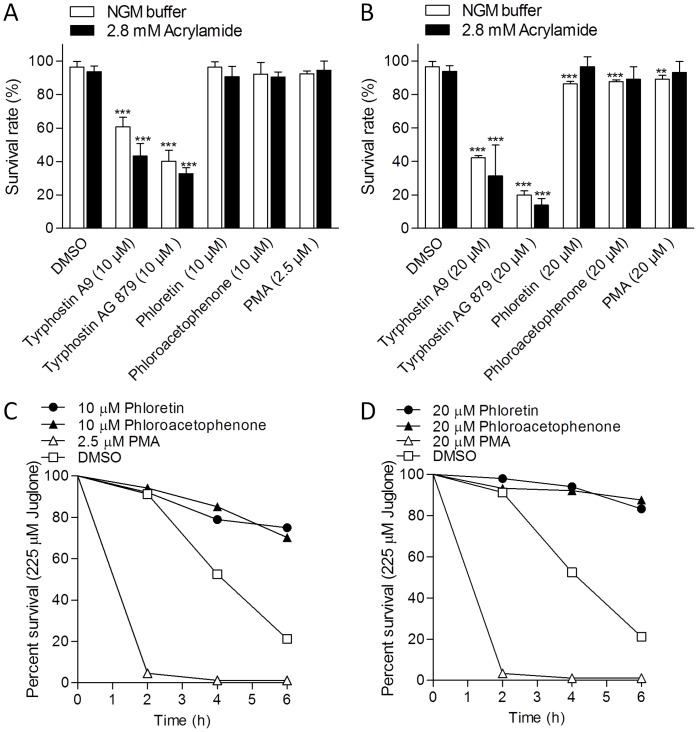
Effects of SKN-1 inhibitors on survival and stress resistance. L4 larval to young adult stage worms were pre-incubated with vehicle control or inhibitors for 1.5 h in 96 well plates. (A-B) Vehicle control (NGM buffer) or 2.8 mM acrylamide was added and survival was measure after 20 h. **P<0.01 and ***P<0.001 compared to control worms (*n = *47–85 worms). (C–D) 225 µM juglone was added and survival was measured for up to 6 h. *P<*0.001 for all inhibitor survival curves relative to DMSO control (*n = *76–121 worms). Values are means ± SEM.

Given the possibility that hit compounds could inhibit gene induction by simply causing general toxicity, we measured survival rates of worms exposed to all five compounds at concentrations required to inhibit *Pgst-4::GFP* in the presence and absence of acrylamide. As shown in Figures 4A and B, tyrphostin A9 and tyrphostin AG879 caused high mortality with and without acrylamide suggesting that these two compounds likely inhibit *Pgst-4::GFP* and *Phsp-16.2* non-specifically by killing worms. None of the other compounds caused significantly more mortality that vehicle controls in the presence of acrylamide (Figures 4A–B). Phloretin, phloroacetophenone, and PMA did significantly reduce survival at 20 µM without acrylamide, but these effects were small (<9%).

Given that *skn-1* promotes longevity and oxidative stress resistance [50], [54], [70], and that SKN-1 is activated by oxidants, we wanted to explore the affects of the low-toxicity inhibitors on resistance to the pro-oxidant juglone, which is lethal to worms at concentrations above 38 µM [58]. PMA dramatically increased sensitivity of worms to juglone (*P<*0.001), consistent with what we expect for compounds that inhibit SKN-1 (Figures 4C–D). Alternatively, phloroacetophenone and phloretin decreased sensitivity to juglone (*P<*0.001) suggesting that they may inhibit *Pgst-4::GFP* by interacting and detoxifying juglone directly. The finding that phloroacetophenone and phloretin did not inhibit induction of *Pgst-4::GFP* when activated by acrylamide (Figure 3) is also consistent with this hypothesis. Acrylamide is used at much greater concentrations (2.8–7.0 mM) than either juglone (38 µM) or hit compounds (5–20 µM) and therefore is in such molar excess (>300-fold) that we expect it to avoid being consumed by test compounds in solution.

### PMA Suppresses gst-4 Expression in Many Tissues

Given that PMA inhibited activation of *gst-4* by xenobiotics and impaired resistance to oxidative stress (Figures 3–4), we characterized it further by determining where it inhibits expression of *Pgst-4::GFP*. Acrylamide broadly induces *Pgst-4::GFP* expression *via* SKN-1 [62]. As shown in Figure S3, 2.5 µM PMA suppressed acrylamide-induced *Pgst-4::GFP* expression broadly in many tissues indicating that its actions are not limited to specific tissues. Importantly, we observed that chronic PMA treatment reduced motility and led to the formation of ‘vacuole-like’ structures, particularly in or near the intestine (Figure S3C). The intestine is a key site of the SKN-1-mediated detoxification program [50], [51]. To determine if the vacuoles caused by PMA might generally inhibit reporter expression in the intestine, we tested its affects on two other GFP reporters expressed in the intestine that are not regulated by SKN-1, one is expressed constitutively (*Pvha-6::GFP)* and the other is *Phsp-16.2::GFP*. Incubation with 20 µM PMA for 20 h had no effect *Pvha-6::GFP* or *Phsp-16.2::GFP* confirming that its inhibition of *Pgst-4::GFP* is specific (Figure S4).

### Primary uHTS Screen of the MLSMR Library of 364,000 Compounds

**Figure 5:**
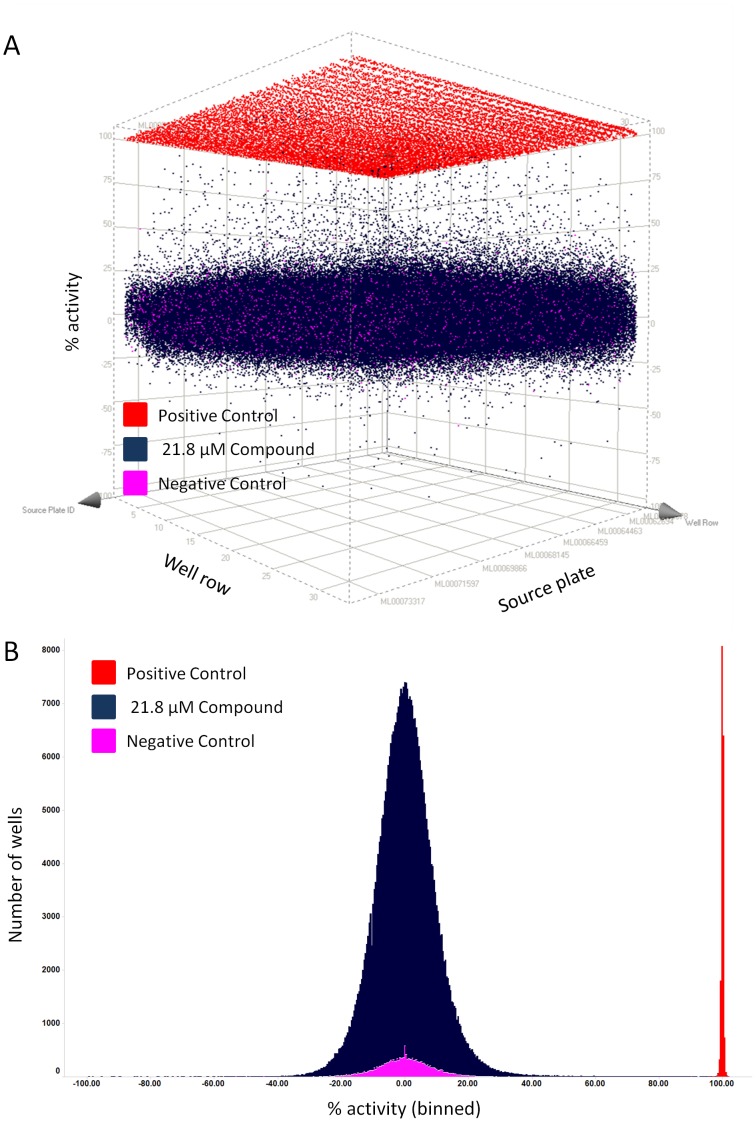
The SKN-1 assay performed well in uHTS. Data from all >364,000 wells of the entire MLSMR library screen are plotted as a 3D scatter of individual points (A) and as a frequency histogram (B) to visualize distribution of % activity. (A and B) Positive controls (red) received no acrylamide and no compound. Negative controls (pink) received acrylamide but no compound. Compound wells (dark blue) received acrylamide and compound (21.8 µM). All wells received worms and buffer. Data were normalized in each plate so that the average GFP/RFP signals for negative and positive controls are 0 and 100%, respectively. Therefore, 100% activity corresponds to complete inhibition of *Pgst-4::GFP* induction, 0% activity corresponds to no inhibition of *Pgst-4::GFP* induction, and a negative % activity corresponds to greater induction of *Pgst-4::GFP*. (A) A 3D scatter plot of % activity (y-axis) for each well versus well row (x-axis) and source plate (z-axis) shows that the majority of compounds (dark blue) had activities between −25% and 25%, similar to the negative control wells that received acrylamide but no compound (pink). Note that many compounds reduced GFP/RFP ratios between 35 and 100%. (B) The frequency histogram was generated by dividing the entire range of % activity of the screening campaign (from −235% to 102%) into 845 equal “bins”. The total number of wells that fell within each bin was then plotted on the y-axis versus % activity. The % activity of compounds (dark blue) forms a normal distribution overlapping with the negative controls (pink). (A–B) Note that 16 data points with % activity less than −100 are not shown in either of the plots; these compounds could have either further activated *Pgst-4::GFP*, strongly reduced *Pdop-3::RFP*, or were fluorescent artifacts.

To validate our platform for uHTS and to identify more SKN-1 inhibitor lead compounds, we applied the assay parameters used with the LOPAC library to screen the entire MLSMR library of ∼364,000 compounds at a final concentration of 21.8 µM (PubChem AID# 624304). This screen used 272 1536-well plates and was accomplished in five weeks at a rate of roughly 50 plates per week. The average Z-factor and signal to background ratios for the 272 plates were 0.73 and 38.6, respectively. A 3D scatter plot of all data from individual wells (Figure 5A) shows that separation of negative and positive controls (with and without acrylamide, respectively) was extremely reproducible and that several compounds reduced GFP/RFP ratio by 35–100% (35–100% activity). A frequency histogram of the same data shows that % activities of compounds are normally distributed and overlap with negative controls (Figure 5B), which is characteristic of a reproducible high throughput screen. At a low stringency inhibition of >35% for GFP/RFP induction and less than a 50% change in RFP relative to negative controls, there were 1795 hit compounds (∼0.5% hit-rate). After cheminformatic filtering to remove known PAINS (Pan Assay Interference Compounds) and promiscuous compounds, 1381 were re-ordered from the MLSMR managed by Evotec (South San Francisco, CA) and retested at six doses in triplicate; 364 (26%) were confirmed and retested at 10 doses in triplicate and 128 of these had an IC_50_ less than 10 µM. These confirmed compounds were tested in the secondary heat shock assay and 125 were specific for *Pgst-4::GFP*. Fifty-six compounds representing 13 chemical scaffolds are being taken forward for structure-activity studies. Taken together, these results demonstrate that our whole-animal screen performed well in a large uHTS screen and identified numerous lead compounds.

## Discussion

We have developed and validated an *in vivo,* fluorescent-based 1536-well plate assay for measuring the activity of a specific gene regulatory pathway that is compatible with ultra HTS. Our assay performed well in pilot screens of the SPECTRUM and LOPAC libraries and in an uHTS screen of the entire ∼364,000 compound MLSMR library. Based on these results, our *in vivo* approach should be applicable to screening for compounds that regulate gene regulatory pathways important to human health and disease, as well as nematode parasitology.

### Off-target Mechanisms

Secondary assays with five hit compounds from the pilot screens revealed two non-specific mechanisms of activity that are likely to be commonly encountered in any *C. elegans* screens using inducible transcriptional reporters. The first non-specific mechanism we identified was general toxicity, which we observed with both tyrphostin compounds (Figure 4). These two tyrphostin-class compounds antagonize receptor tyrosine kinases (RTKIs) that regulate growth, metabolism, and differentiation [71]. Obviously, sick or dead animals will not be able to activate inducible transcriptional pathways. In our case, the secondary heat-shock response assay based on *in vivo Phsp-16.2::GFP* fluorescence was also inhibited by both tyrphostins (Figure 3) and manual mortality assays confirmed that toxicity was the likely mechanism (Figure 4). We also expect this secondary reporter assay to identify compounds that generally inhibit transcription or translation. To identify cytotoxicity, luciferase-based ATP or dye-based metabolic assays are used routinely for cultured cells [72], [73] and can be modified for use with *C. elegans* [74].

The second non-specific mechanism of reporter inhibition that we observed was direct interactions between a hit compound and a chemical inducer of *Pgst-4::GFP*. The related phloretin [b-(4-hydroxyphenyl)-1-(2,4,6-trihydroxypropiophenone)] and phloroacetophenone (2′,4′,6′trihydroxyacetophenone-THA) compounds appear to have reduced the bioactivity of juglone. Phloroacetophenone and phloretin have both been reported to have antioxidant activity [75], [76], [77], [78] and likely react with the electrophile juglone [79] directly. Therefore, concentration and reactivity of a chemical inducer must be carefully considered to reduce the chances of direct compound-compound interactions outside of the biological system. We expect that using acrylamide in over 300-fold molar excess of screening compounds will greatly reduce the number of hits with this non-specific mechanism. Using reporters that are induced by non-chemical environmental conditions (e.g., temperature) can also be used to avoid this problem during primary screening. After primary screening with a chemical inducer, secondary screens with distinct chemical inducers (e.g., juglone versus acrylamide), or alternative assays for the bioactivity of the inducer (e.g., toxicity), can be used to help determine if a compound acts on the biological pathway or the inducing chemical.

PMA strongly suppressed acrylamide and juglone-induced *Pgst-4::GFP* induction but not *Phsp-16.2::GFP* induction (Figure 3). PMA was not highly toxic to worms by itself, but it did dramatically sensitize worms to juglone (Figure 4), as would be expected from inhibition of the SKN-1 detoxification pathway. PMA is a potent agonist of protein kinase C (PKC), which functions in early development, immunity, cell division, cell migration, and apoptosis [80], [81], [82]. Although further studies are needed to define a specific mechanism, our results suggest a novel pathway of *gst-4* (and maybe SKN-1) regulation involving PKC in *C. elegans*. Interestingly, PKC was previously reported to activate Nrf2 in mammalian cells [83], [84]. Although PMA displayed many characteristics desired for a SKN-1 inhibitor, the broad and conserved functions of PKC make it a poor candidate for further development. Therefore, our future efforts will focus on developing lead compounds from the MLSMR library.

### Drug Accumulation

Relative to cells in culture, accumulation of many drugs in *C. elegans* can be inadequate because of low permeability through the think cuticle of nematodes [85]. We did not view this as a disadvantage for our SKN-1 inhibitor screen because compounds with poor accumulation in *C. elegans* would likely also have poor accumulation in parasitic nematodes. Alternatively, low permeability is an important consideration in screens for modulators of human pathways. Fortunately, accumulation of structurally diverse drugs has been reported to be enhanced in *C. elegans* strains with mutations in the gene *bus-8* that cause epidermis and cuticle disorganization [86]. Use of these mutations would be expected to improve hit rates in *C. elegans* screens.

### Perspectives

An *in vivo* animal model has yet to be developed that is compatible with automated screening facilities and libraries of over 100,000 compounds. The nematode *C. elegans* has an ideal combination of small size, complexity, simple culture characteristics, and genetic tractability for *in vivo* high-throughput screening. We have validated the first whole-animal based assay for a specific molecular target in an ultra high-throughput screen. We developed this screening platform to identify small molecule inhibitors of the drug detoxification gene master regulator SKN-1 that will serve as probes to study and potentially reverse drug resistance in parasitic nematodes. We expect this same approach to be applicable to numerous nematode and human pathways.

*C. elegans* is one of the best studied and understood metazoans and for the past 30+ years has been a major model for dissecting molecular mechanisms of development, metabolism, behavior, and aging. Our screening platform, for the first time, opens the door for researchers in these fields to apply small molecule screening to the great number of gene regulatory pathways that have been characterized. Many gene regulatory pathways important to human health are conserved in *C. elegans*. Furthermore, *C. elegans* can be engineered to express human transcription factors and promoter-driven fluorescent reporters to screen for modulators of human disease-associated factors.

## Supporting Information

Figure S1 **The heat-shock response assay is stable and tolerant of DMSO.** (A) QV65 worms were heat-shocked at 35°C for 1 or 2 h and then recovered at 20°C; control plates were setup similarly but were maintained at 20°C without heat-shock (*n = *384 wells). Values are means ± SEM. (B) QV65 worms were dispensed into a 384-well plate with different DMSO concentrations and heat-shocked at 35°C for 1 h followed by recovery at 20°C for 5 h; values are relative to a control plate that was maintained at 20°C without heat-shock (*n = *64 wells). The corresponding Z’ scores of individual treatments are listed inside each bar. Values are means+SEM. Fluorescence is significantly different from controls at all DMSO concentrations tested (*P<*0.01).
(TIF)

Figure S2 **Endogenous SKN-1 target genes are suppressed by inhibitors.** Wild-type worms were first incubated with inhibitor compounds at 20 µM for 1.5 h followed by 2.8 mM acrylamide (A) or 38 µM juglone (B) for 4 h. Relative mRNA levels of SKN-1 target genes were then measured with real-time RT-PCR. The values are means plus standard errors (*n = *5 populations of 100 to 300 worms). **P<0.01 and ***P<0.001 compared to control worms. †*P<*0.05, ††*P<*0.01, and †††*P<*0.001 compared to acrylamide or juglone treated worms.
(TIF)

Figure S3 **PMA broadly inhibits** ***Pgst-4::GFP*** **expression.** Representative differential interference contrast (left) and fluorescence (right) micrographs of VP596 L4 larval to young adult stage worms treated with vehicle control, DMSO (A) or 2.5 µM PMA (B) followed by 2.8 mM acrylamide for 20 h. The images of *Pgst-4::GFP* expression were taken at different focal planes or areas to highlight specific tissues. (C) Arrows mark structures with the appearance of vacuoles that were commonly observed in PMA-treated worms. Scale bars = 50 µm
(TIF)

Figure S4 **PMA does not generally inhibit intestinal GFP expression.** Representative differential interference contrast (left) and fluorescence (right) micrographs of: (A) *Pvha-6::GFP* expressing worms treated with vehicle control (DMSO) or 20 µM PMA for 20 h or (B) *Phsp-16.2::GFP* expressing worms treated with vehicle control (DMSO) or 20 µM PMA for 1.5 h, then exposed to control (20°C) or heat-shock (35°C) temperature for 1 h followed by 5 h recovery at 20°C. Scale bars = 50 µm.
(TIF)

File S1 **This file contains three Supplementary Tables S1–S3.** Table S1, Hit compounds from the Spectrum Library; Table S2, Induction of *Pgst-4::GFP* by acrylamide in 1536-well plates; and Table S3, Hit compounds from the LOPAC library.
(DOCX)
