# Supplementary figures and images for: An Ultra High-Throughput, Whole-Animal Screen for Small Molecule Modulators of a Specific Genetic Pathway in Caenorhabditis elegans
Source: PLoS One. 2013 Apr 29;8(4):e62166. doi: 10.1371/journal.pone.0062166 (PMC3639262; doi:10.1371/journal.pone.0062166)

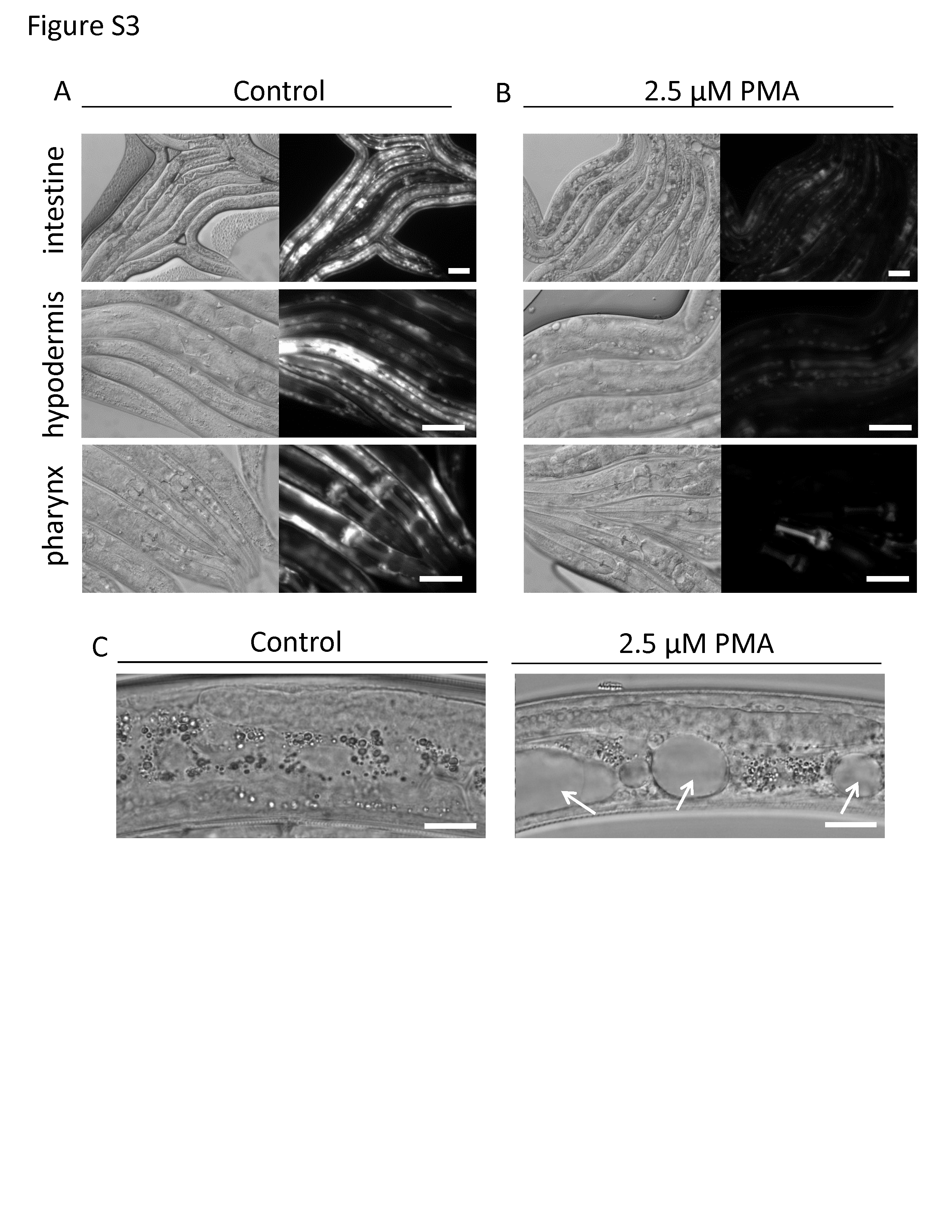

Supplement: Figure S3 — PMA broadly inhibits Pgst-4::GFP expression. Representative differential interference contrast (left) and fluorescence (right) micrographs of VP596 L4 larval to young adult stage worms treated with vehicle control, DMSO (A) or 2.5 µM PMA (B) followed by 2.8 mM acrylamide for 20 h. The images of Pgst-4::GFP expression were taken at different focal planes or areas to highlight specific tissues. (C) Arrows mark structures with the appearance of vacuoles that were commonly observed in PMA-treated worms. Scale bars = 50 µm (TIF) [file pone.0062166.s003.tif]

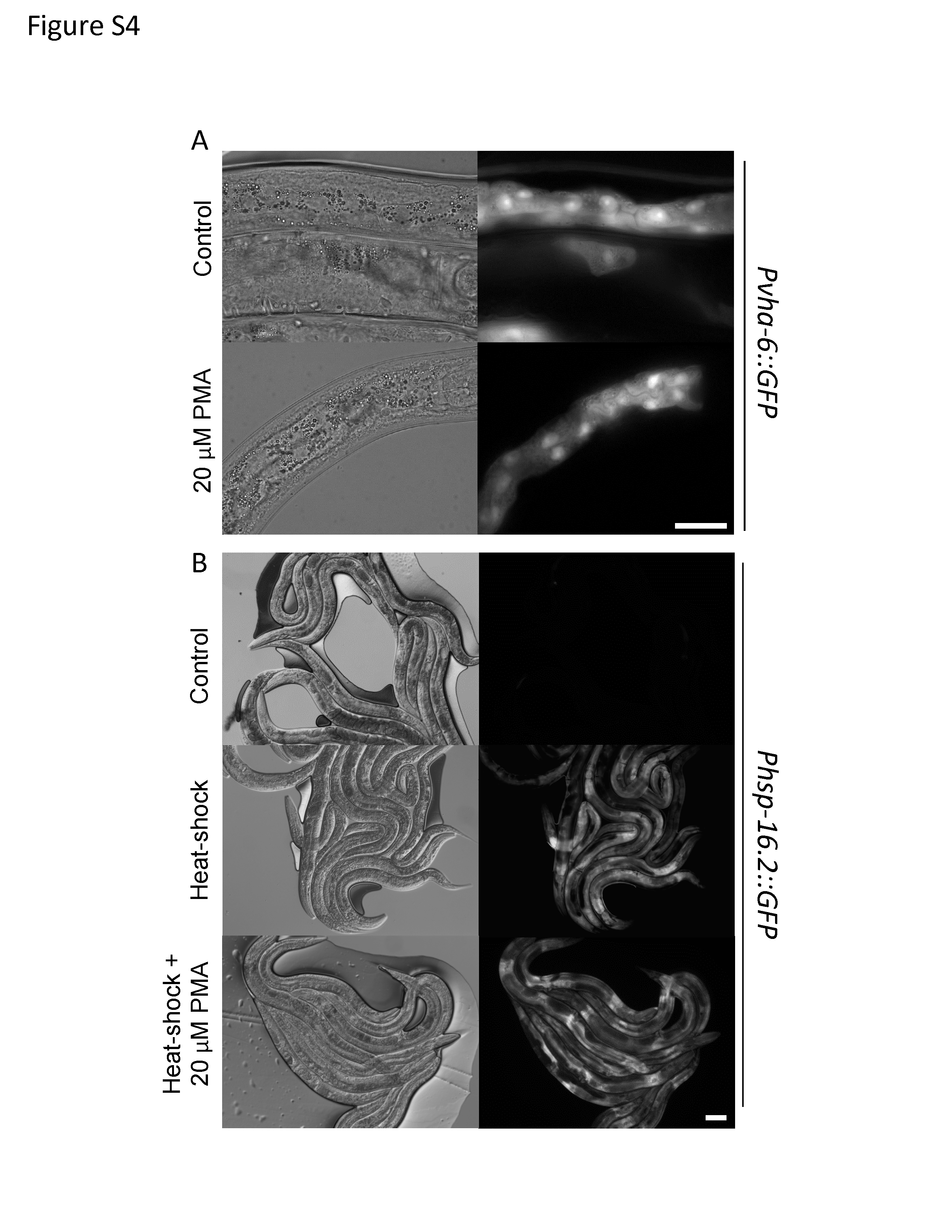

Supplement: Figure S4 — PMA does not generally inhibit intestinal GFP expression. Representative differential interference contrast (left) and fluorescence (right) micrographs of: (A) Pvha-6::GFP expressing worms treated with vehicle control (DMSO) or 20 µM PMA for 20 h or (B) Phsp-16.2::GFP expressing worms treated with vehicle control (DMSO) or 20 µM PMA for 1.5 h, then exposed to control (20°C) or heat-shock (35°C) temperature for 1 h followed by 5 h recovery at 20°C. Scale bars = 50 µm. (TIF) [file pone.0062166.s004.tif]
