# Supplementary material for: An Ultra High-Throughput, Whole-Animal Screen for Small Molecule Modulators of a Specific Genetic Pathway in Caenorhabditis elegans
Source: PLoS One. 2013 Apr 29;8(4):e62166. doi: 10.1371/journal.pone.0062166 (PMC3639262; doi:10.1371/journal.pone.0062166)
Supplement: File S1 — This file contains three Supplementary Tables S1–S3. Table S1, Hit compounds from the Spectrum Library; Table S2, Induction of Pgst-4::GFP by acrylamide in 1536-well plates; and Table S3, Hit compounds from the LOPAC library. (DOCX) [file pone.0062166.s005.docx]

Table S1. Hit compounds from the Spectrum Library

|  |  | % Activity (% inhibition of *Pgst-4::GFP/Pdop-3::RFP*) | | |  |  |
| --- | --- | --- | --- | --- | --- | --- |
| Compound ID | Name | 10 µM | 20 µM | 40 µM | Structure | Comments |
| 00300554 | Phloretin | 45.4 | 60.8 | 51.8 | 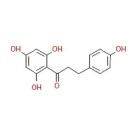 | Antioxidant |
| 00300604 | Phloracetophenone | 40.8 | 51.6 | 53.9 | 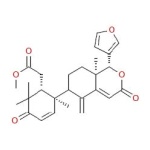 | Antioxidant |
| 00210205 | Cianidanol | 42.5 | 43.6 |  | 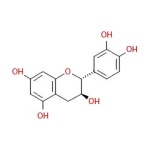 | Procollagen production inhibitor, hepatoprotectant |
| 01500629 | Flopropione | 47.0 |  | 52.6 | 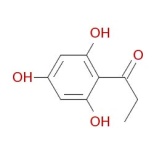 | Antispasmodic |
| 01503806 | Homidium bromide | 83.6 |  |  | 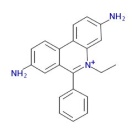 | Anti-protozoal, intercalcate with DNA |
| 01500644 | Phenylmercuric acetate | 47.4 | 48.2 | 41.3 | 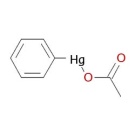 | Antifungal |
| 01502209 | Phenethyl caffeate (cape) |  | 46.2 |  | 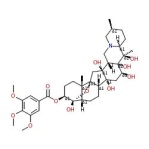 | Anti-neoplastic, anti-inflammatory, immunomodulator, NFkB blocker |
| 00200759 | Iretol |  | 42.7 |  | 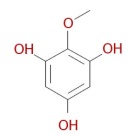 | Unknown function |
| 01505331 | 3,3'-Diindolylmethane |  | 45.8 |  | 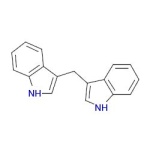 | Apoptosis inducer |
| 00203008 | Juarezic acid |  | 49.0 |  | 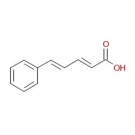 | Unknown function |
| 00330071 | Lindane |  | 41.4 |  | 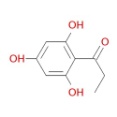 | Insecticide |
| 00240826 | Purpurogallin-4-carboxylic acid |  | 49.6 |  | 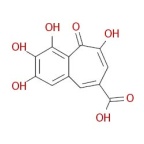 | Antioxidant |
| 02300165 | Amiodarone hydrochloride |  | 43.4 |  | 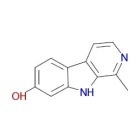 | Adrenergic agonist, coronary vasodilator, Ca^2+^ channel blocker |
| 00330062 | Temefos |  | 42.9 |  | 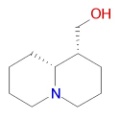 | Insecticide |
| 01500184 | Chlorpromazine |  | 46.8 |  | 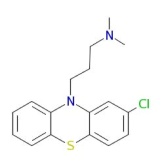 | Antiemetic, antipsychotic |
| 01500206 | Colistimethate sodium |  | 43.2 | 47.2 | 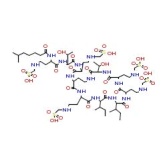 | Antibacterial |
| 01500233 | Dextromethorphan hydrobromide |  | 40.8 |  | 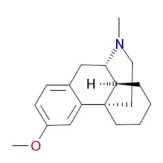 | Anti-tussive |
| 01500235 | Dibenzothiophene |  | 44.6 |  | 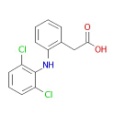 | Keratolytic |
| 01501147 | Sulfamonomethoxine |  | 40.2 |  | 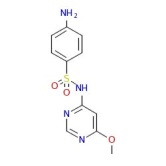 | Antibacterial |
| 01501215 | Ketoprofen |  | 43.9 |  | 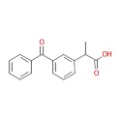 | Anti-inflammatory |
| 00310035 | Sanguinarine sulfate |  | 47.7 |  | 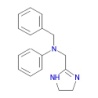 | Anti-neoplastic, anti-plaque agent |
| 01500476 | Phenelzine sulfate |  | 46.5 |  | 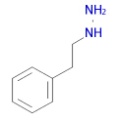 | Anti-depressant |
| 01500178 | Chlorocresol |  | 46.1 |  | 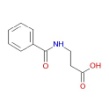 | Anti-infectant |
| 01500209 | Cresol |  | 42.8 |  | 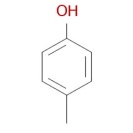 | Anti-infectant |
| 00240944 | Haematoxylin pentaacetate |  | 41.9 | 42.8 | 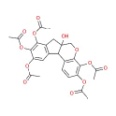 | Unknown function |
| 00330001 | Dactinomycin |  | 42.8 |  | 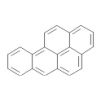 | Anti-neoplastic, intercalating agent |
| 01503298 | Dipyrone |  | 47.2 | 48.3 | 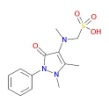 | Analgesic, antipyretic |
| 01503650 | Nabumetone |  | 41.3 |  | 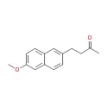 | Anti-inflammatory |
| 01500629 | Flopropione |  | 56.3 |  | 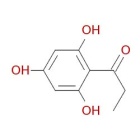 | Antispasmodic |
| 01505319 | Clopidol |  | 42.3 |  | 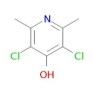 | Aoccidiostat, antiplatelet |
| 00200111 | Theaflavin |  |  | 44.6 | 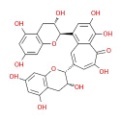 | Antioxidant |
| 01502006 | Carprofen |  |  | 42.7 | 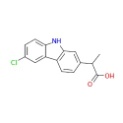 | Anti-inflammatory, analgesic |
| 01500860 | s-Isocorydine (+) |  |  | 48.6 | 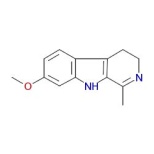 | Aedative, cholinergic |
| 01600025 | 2,6-Dihydroxy-4-methoxytoluene |  |  | 46.3 | 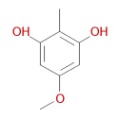 | Unknown function |
| 00211475 | 4'-Methoxychalcone |  |  | 43.0 | 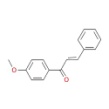 | Unknown function |
| 00210515 | Pyrogallin |  |  | 44.4 | 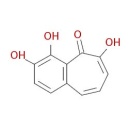 | Anti-infectant |
| 01504021 | Lupinine |  |  | 41.7 | 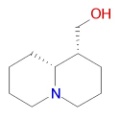 | Anti-feedant, anti-inflammatory, oxytoxic |
| 01503223 | Pararosaniline pamoate |  |  | 52.3 | 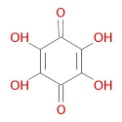 | Anthelmintic, anti-schistosomal |
| 01500455 | Oxyphenbutazone |  |  | 41.2 | 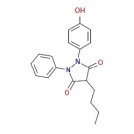 | Anti-inflammatory |
| 01500521 | Pyrvinium pamoate |  |  | 47.3 | 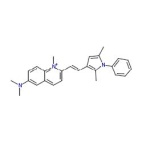 | Anthelmintic |
| 01500991 | Glycyrrhizic acid, ammonium salt |  |  | 45.2 | 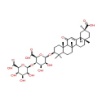 | β-hydroxysteroid dehydrogenase inhibitor, anti-inflammatory, expectorant, anti-haemorrhagic, anti-HIV |
| 01500584 | Tranylcypromine sulfate |  |  | 42.1 | 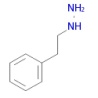 | Antidepressant, MAO inhibitor |
| 01504167 | Ursinoic acid |  |  | 42.5 | 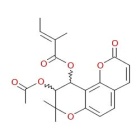 | Unknown function |
| 01504216 | Zoxazolamine |  |  | 41.3 | 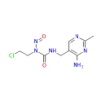 | Muscle relaxant, antirheumatic |
| 01504244 | Betamipron |  |  | 42.3 | 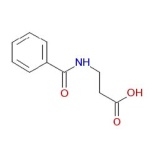 | Sweetener |
| 00210206 | Epicatechin |  |  | 43.6 | 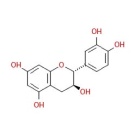 | Antioxidant |
| 00205113 | Epigallocatechin |  |  | 42.0 | 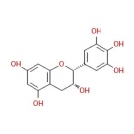 | Antioxidant |
| 01503650 | Nabumetone |  |  | 42.1 | 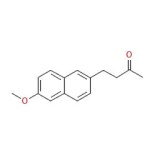 | Anti-inflammatory |
| 01500133 | Azathioprine |  |  | 42.3 | 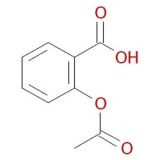 | Immunosuppressant, antineoplastic, antirheumatic |
| Compound IDs are the Spectrum Library order numbers. Compounds were included in this list if they had over 40% activity and RFP values within three standard deviations of control wells at any concentration; wells that did not meet these criteria are blank. | | | | | | |

Table S2. Induction of *Pgst-4::GFP* by acrylamide in 1536-well plates

| No. of worms/well | S/B | Z factor |
| --- | --- | --- |
| 10-15 | 20.11±3.78 | 0.38 |
| 15-20 | 21.06±3.55 | 0.45 |
| 20-25 | 20.87±2.72 | 0.56 |
| 30-35 | 18.92±1.47 | 0.72 |
| S/B = signal to background. 1536-well plates. *n* = 768 wells per treatment. | | |

Table S3. Hit compounds from the LOPAC library

|  |  | % Activity (% inhibition of *Pgst-4::GFP/Pdop-3::RFP*) | | | | | | | | |  |  |
| --- | --- | --- | --- | --- | --- | --- | --- | --- | --- | --- | --- | --- |
| Compound ID | name | 5 µM | | | 10 µM | | | 20 µM | | | Structure | Comments |
| T-182 | Tyrphostin A9 | 90.4 | 91.4 | 90.2 | 83.1 | 88.6 | 93.0 | 89.1 | 88.4 | 90.7 |  | Selective PDGF tyrosine kinase receptor inhibitor |
| P 8139 | Phorbol 12-myristate 13-acetate | 88.5 | 84.9 | 89.1 | 85.0 | 90.7 | 81.4 | 87.6 | 79.9 | 91.3 |  | Activates protein kinase C in vivo and in vitro; strong NO promoter; promotes expression of iNOS in cultured hepatocytes;T lymphocyte activator |
| T 2067 | Tyrphostin AG 879 | 57.1 | 42.2 |  | 89.9 | 80.8 | 84.0 | 78.6 | 73.8 | 82.9 |  | Tyrosine kinase nerve growth factor receptor (TrkA) inhibitor; inhibits 140 trk protooncogene and HER-2 |
| Z2777 | Ziprasidone hydrochloride monohydrate | 70.4 | 40.9 |  | 50.1 | 40.3 | 48.4 |  |  |  |  | Atypical antipsychotic; FDA approved for the treatment of schizophrenia. |
| A8423 | Amiodarone hydrochloride |  |  |  |  | 53.4 |  | 57.3 | 52.9 | 56.4 |  | Alpha and beta adrenoceptor agonist; inhibits binding of 1,4-dihydropyridine to L-type Ca^2+^ channels; coronary vasodilator |
| S3442 | SB 216763 |  |  |  |  | 45.9 | 42.5 |  |  |  |  | Potent, selective, cell permeable inhibitor of glycogen synthetase kinase-3 (GSK-3). |
| C1671 | Chlorprothixene hydrochloride | 52.9 |  |  | 48.6 |  |  |  |  |  |  | D2 dopamine receptor antagonist; blocks a subset of GABA-A receptors in rat cortex that is also blocked by clozapine |
| C8138 | Chlorpromazine hydrochloride |  | 44.0 |  |  | 51.2 | 50.3 |  |  |  |  | Dopamine receptor antagonist; anti-emetic; antipsychotic |
| L9756 | Levamisole hydrochloride |  | 51.7 | 49.7 |  |  |  | 53.4 | 44.6 | 70.1 |  | Inhibits multiple mammalian alkaline phosphatases |
| I8898 | Ivermectin | 73.0 |  | 74.4 |  |  | 61.4 |  |  |  |  | Positive allosteric modulator of alpha7 neuronal nicotinic acetylcholine receptor; also modulates glutamate-GABA-activated chloride channels |
| M2525 | Mianserin hydrochloride |  |  |  |  | 44.4 |  |  | 41.4 | 45.3 |  | Serotonin receptor antagonist |
| M-149 | Methiothepin mesylate salt |  | 45.2 | 63.8 | 51.4 |  | 51.8 |  |  |  |  | 5-HT1 Serotonin receptor antagonist; blocks serotonin autoreceptors |
| O-111 | Octoclothepin maleate salt | 49.5 | 53.6 |  |  |  |  | 46.0 | 57.1 | 45.2 |  | D2 Dopamine receptor antagonist; serotonin receptor antagonist |
| S8442 | SU 5416 |  |  |  | 42.4 |  |  |  |  |  |  | Potent and selective VEGFR PTK inhibitor; inhibits VEGF-induced angiogenesis |
| R8875 | Rotenone |  |  |  |  |  | 54.1 | 54.7 |  | 69.6 |  | Inhibitor of mitochondrial electron transport |
| T1512 | Tetramisole hydrochloride |  |  |  |  |  |  | 54.2 | 64.0 | 43.8 |  | Inhibits various mammalian alkaline phosphatases (i.e., liver, kidney, placenta, bone and tumor) |
| T9025 | Thioridazine hydrochloride |  |  |  |  |  |  | 44.1 |  | 40.6 |  | Dopamine receptor antagonist; Ca^2+^ channel antagonist; antipsychotic |
| D5676 | N-Desmethylclozapine |  |  |  |  |  |  |  |  | 50.5 |  | Major metabolite of clozapine; potent 5-HT2 serotonin receptor antagonist and a ligand for the cloned 5-HT6 and 5-HT7 serotonin receptors. Also an allosteric potentiator of M1 muscarinic receptors |
| M3184 | MG 624 |  |  |  |  |  |  | 43.0 |  |  |  | Nicotinic acetylcholine receptor antagonist; selectively inhibits alpha-bungarotoxin sensitive receptors that contain the α7 subunit |
| T3146 | Trimipramine maleate salt |  |  |  |  | 40.5 |  |  |  |  |  | Serotonin reuptake inhibitor that also blocks norepinephrine reuptake; antidepressant |
| S5890 | Sanguinarine chloride hydrate |  |  |  |  |  |  |  |  | 49.8 |  | Inhibitor of Mg^2+^ and Na^+^/K^+^-ATPase; isolated from the leaves and stems of Macleaya cordata and microcarpa |
| N0630 | Niflumic acid |  |  |  |  |  |  |  |  | 41.1 |  | Selective prostaglandin H synthase 2 (PGHS-2) inhibitor |
| L-106 | Loxapine succinate salt |  |  |  |  |  |  |  | 42.1 |  |  | Dibenzoxazepine antipsychotic agent |
| L9664 | Loratadine |  |  |  |  |  |  |  | 45.7 |  |  | H1 Histamine receptor antagonist |
| M1404 | Nocodazole |  |  |  |  |  | 65.3 |  |  |  |  | Disrupts microtubules by binding to β-tubulin |
| B2515 | L-Buthionine-sulfoximine |  |  |  |  |  |  |  | 40.0 |  |  | Glutathione synthesis inhibitor |
| F6886 | Forskolin |  |  |  |  |  |  |  | 40.0 |  |  | Activates adenylate cyclase; antihypertensive and vasodilator; isolated from *Coleus forskohlii* |

Compound IDs are Sigma-Aldrich order numbers. The LOPAC library was screened at three concentrations, in triplicate. Compounds were included in this list if they had over 40% activity and RFP values within 50% of control wells in at least one replicate; wells that did not meet these criteria are blank.
